# Spatial clustering of measles cases during endemic (1998–2002) and epidemic (2010) periods in Lusaka, Zambia

**DOI:** 10.1186/s12879-015-0842-y

**Published:** 2015-03-10

**Authors:** Jessie Pinchoff, James Chipeta, Gibson Chitundu Banda, Samuel Miti, Timothy Shields, Frank Curriero, William John Moss

**Affiliations:** Department of International Health, Johns Hopkins University Bloomberg School of Public Health, Baltimore, MD USA; Department of Paediatrics and Child Health, University of Zambia School of Medicine, P.O. Box 50110, Lusaka, Zambia; Department of Epidemiology, Johns Hopkins University Bloomberg School of Public Health, Baltimore, MD USA

**Keywords:** Measles, Epidemiology, Zambia, Surveillance, Spatial Clustering

## Abstract

**Background:**

Measles cases may cluster in densely populated urban centers in sub-Saharan Africa as susceptible individuals share spatially dependent risk factors and may cluster among human immunodeficiency virus (HIV)-infected children despite high vaccination coverage.

**Methods:**

Children hospitalized with measles at the University Teaching Hospital (UTH) in Lusaka, Zambia were enrolled in the study. The township of residence was recorded on the questionnaire and mapped; SaTScan software was used for cluster detection. A spatial-temporal scan statistic was used to investigate clustering of measles in children hospitalized during an endemic period (1998 to 2002) and during the 2010 measles outbreak in Lusaka, Zambia.

**Results:**

Three sequential and spatially contiguous clusters of measles cases were identified during the 2010 outbreak but no clustering among HIV-infected children was identified. In contrast, a space-time cluster among HIV-infected children was identified during the endemic period. This cluster occurred prior to the introduction of intensive measles control efforts and during a period between seasonal peaks in measles incidence.

**Conclusions:**

Prediction and early identification of spatial clusters of measles will be critical to achieving measles elimination. HIV infection may contribute to spatial clustering of measles cases in some epidemiological settings.

## Background

Despite a 74% decline in global measles deaths between 2000 and 2010 [1,2], measles remains a significant cause of mortality in children younger than five years of age. In the African region, there were an estimated 139300 measles deaths in 2010 [2]. The Measles and Rubella Initiative developed a joint strategic plan to reduce measles deaths by strengthening routine immunization, providing a second dose of measles vaccine through supplemental immunization activities (SIAs) in the form of mass vaccination campaigns, enhanced surveillance with laboratory confirmation of suspected cases, and appropriate case management [3]. However, measles elimination remains challenging in sub-Saharan Africa where obstacles to achieving high coverage with two doses of measles vaccine include weak routine immunization services, high force of infection in densely populated urban settings, conflict and civil unrest, and limited financial resources [4,5].

Infectious diseases such as measles tend to cluster geographically where susceptible individuals reside in close proximity. As a result, spatial analyses are important to detect and predict the spatial and temporal patterns of infectious diseases [6-9]. For pathogens directly transmitted from person-to-person, such as measles virus, disease outbreaks may cluster in urban areas where the susceptible population is large and dense [10]. High vaccination coverage may interrupt these processes over large geographical areas [11].

Potentially impacting the spatial clustering of measles are regions of high human immunodeficiency virus (HIV) prevalence, particularly in urban areas. Recent measles outbreaks in South Africa were reported to be associated with HIV infection [6]. There are several reasons HIV-infected children may play a role in maintaining measles virus transmission [12-15]. HIV-infected mothers may have defective transfer of IgG antibody across the placenta, resulting in lower levels of protective antibodies in the infant and enlarging the period of susceptibility to measles virus infection prior to routine immunization [16,17]. HIV-infected children may not have an adequate primary response to measles immunization and may lose immunity with progressive immunosuppression, thus remaining susceptible to measles virus despite immunization [18], with immunity not restored by antiretroviral therapy [19]. Children with defective cell-mediated immunity may not develop the characteristic measles rash so infection may go unrecognized [18,20]. And HIV-infected children may have prolonged shedding of measles virus increasing the period of infectivity and the spread of measles virus to secondary contacts [21].

Clustering of measles in both space and time has been identified at the country and regional levels in South Africa and Niger [6,22,23], but no studies have examined clustering in an urban setting of sub-Saharan Africa. Spatial-temporal clustering of measles was investigated during the 2010 outbreak in Lusaka, Zambia, and specifically whether HIV-infected children with measles were spatially clustered during the outbreak. Spatial-temporal clustering of measles cases among HIV-infected children was further explored during an endemic period (1998–2002) prior to national SIAs.

## Methods

### Study site

The University Teaching Hospital (UTH) is the largest public hospital in Lusaka, Zambia with a 415-bed Paediatrics and Child Health wing. Zambia has one of the highest HIV prevalence rates in sub-Saharan Africa and was prone to seasonal outbreaks of measles prior to the first national mass vaccination campaign in 2003 [4]. In Zambia, 111 measles cases were reported in 2008 and only 26 cases in 2009. However, an outbreak resulting in 15736 reported cases occurred in 2010 [13,22]. Measles SIAs were conducted in 2007 and 2010.

### 1998-2002 endemic period

During the endemic period between 1998 and 2002, a prospective observational study of measles in HIV-infected and uninfected children hospitalized was conducted at the UTH [24]. Clinical and demographic data were collected at enrollment and included date of hospitalization and the township in which the child resided. Plasma was tested for antibodies to HIV by enzyme immunoassay (Organon Tecknika, Boxtel, The Netherlands). Plasma levels of HIV RNA were quantified by reverse transcriptase polymerase chain reaction assay (Amplicor HIV-1 Monitor v 1.5, Roche Molecular Systems, Branchburg, NJ) in children with antibodies to HIV. Children were classified as HIV-infected if HIV-1 RNA was detected in plasma. Measles virus infection was confirmed by detection of measles virus-specific IgM in plasma by EIA (Wampole Laboratories, Cranbury, NJ). Written informed consent was obtained from parents or guardians of study children. The study protocol was approved by institutional review boards at the Johns Hopkins University Bloomberg School of Public Health and the University Teaching Hospital, Lusaka, Zambia.

### 2010 measles outbreak

Clinical and epidemiological characteristics were retrospectively extracted from available patient records for all children younger than 16 years of age admitted to the UTH with a diagnosis of measles between January and December 2010. A detailed case assessment questionnaire was used to extract patient data, including date of hospitalization, measles vaccination history, township of residence and HIV infection status. For children older than 18 months of age, two independent enzyme immunoassays (an Abbott Determine rapid test kit [Alere Determine™ HIV-1/2 Ag/Ab Combo, Orgenics Ltd, Yavne Israel] followed by a confirmatory test with Genie II [Uni-Gold™ HIV, Trinity Biotech, Ireland] for positive rapid tests) were used to establish HIV infection. For infants younger than 18 months of age, HIV infection was confirmed by detection of HIV DNA by polymerase chain reaction (PCR) (Amplicor HIV-1 Monitor v 1.5, Roche Molecular Systems, Branchburg, NJ). HIV seropositive children younger than 18 months of age for whom HIV DNA testing was not performed were considered HIV exposed. Children with unknown HIV infection status were excluded from spatial analyses (N = 88, 7.5%). Measles was diagnosed clinically and was confirmed by detection of IgM antibodies to measles virus by EIA (Wampole Laboratories, Cranbury, NJ). Date of hospitalization was used as a proxy for date of illness. Ethical approval was obtained by the University of Zambia (UNZA) Research Ethics Committee. The authors assert that all procedures contributing to this work comply with the ethical standards of the relevant national and institutional committees on human experimentation and with the Helsinki Declaration of 1975, as revised in 2008.

### Spatial analysis

Hospitalized children with clinical or laboratory confirmed measles, known HIV infection or seropositive status, and who resided within Lusaka were included in the spatial analysis. An ArcGIS map of the townships of Lusaka was obtained from the Central Office of Statistics, Lusaka, Zambia for 2000 and for 2010. For 2010, incidence was calculated as the number of measles virus-infected children per township divided by the total population for that township obtained from the Central Statistics Office on the assumption that few measles cases occurred in adults. Cases were reported per day but were aggregated to the weekly level for analysis. A map was created showing the population density of Lusaka townships as persons per square kilometer. Three separate space-time cluster detection analyses were performed. The first compared HIV-infected to HIV-uninfected children to measure clustering of measles cases among HIV-infected children. This was conducted for both the 1998–2002 endemic and 2010 epidemic periods. The third space-time cluster detection analysis was conducted using all reported measles cases (independent of HIV infection status) to describe spatial clustering of measles cases during the 2010 epidemic period.

Case and control files, consisting of a unique identification number for the township of each child and the date of hospitalization, were constructed for HIV-infected (cases) and uninfected (controls) children hospitalized with measles. Children in whom HIV infection was not laboratory-confirmed were considered uninfected. The space-time cluster detection analyses were performed using SaTScan version 5.1 [25]. SaTScan is a method for spatial, temporal and space-time cluster detection analysis that has been used in a wide variety of applications [9,26,27]. For the current application, case and control information were aggregated to the spatial township level and clusters were sought using a 7-day temporal scale. The expected number of cases was based on the distribution of all hospitalized measles cases. Statistical significance for identified clusters was based on Monte Carlo simulation using the Bernoulli probability model option in SaTScan, which is most appropriate for case–control data, adjusting for township population density [25]. Significance for the 2010 outbreak analyses employed a permutation scheme within SaTScan as temporal changes in township population would be negligible over the one-year time frame [25,28].

## Results

### 1998-2002 endemic period

**Figure 1:**
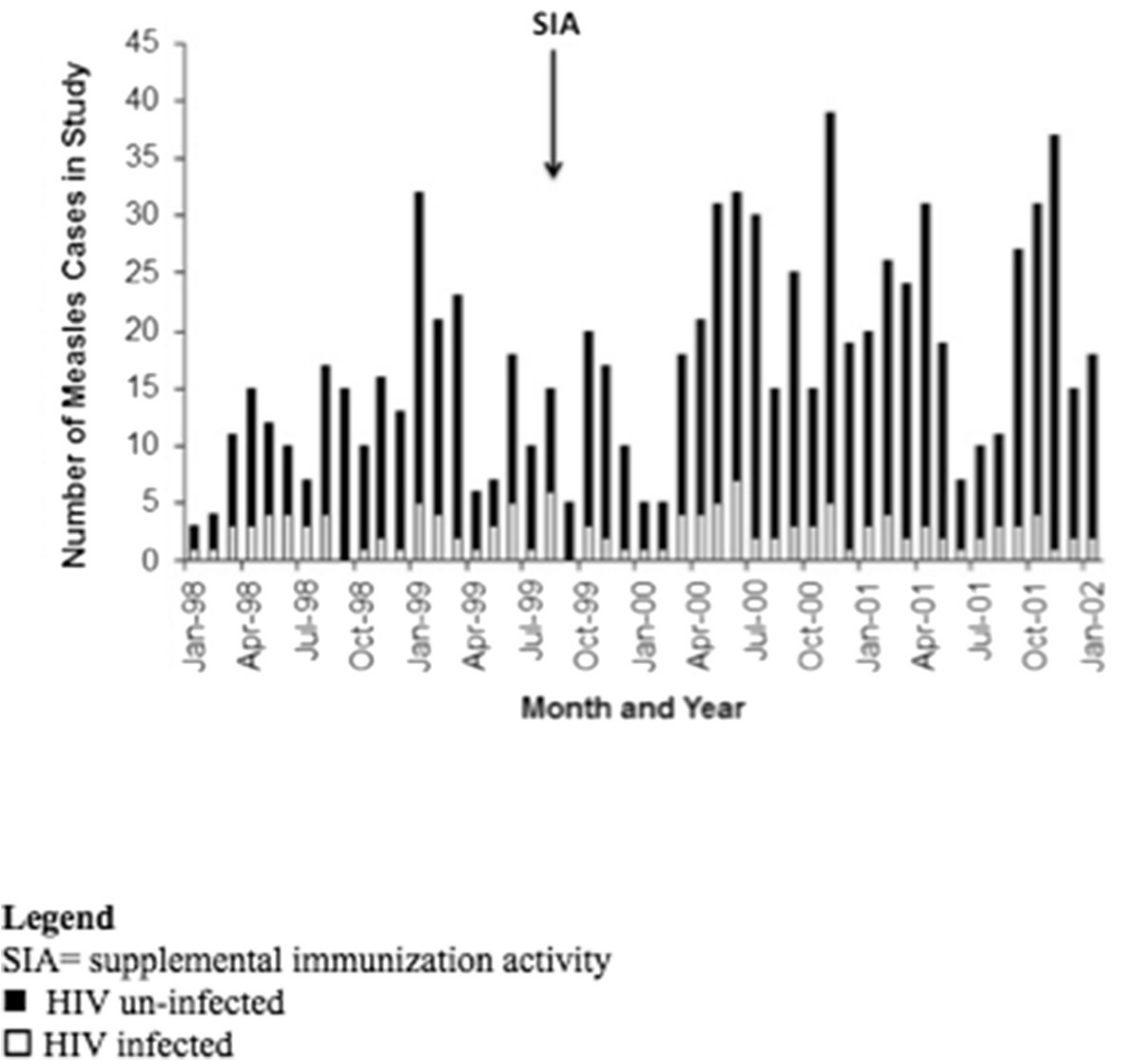
**Time-series of hospitalized children with measles, Lusaka, Zambia and included in the analysis of space-time clustering, 1998–2002.**

For the endemic period between January 1998 and January 2002, 1323 hospitalized children with suspected measles were enrolled. Of these, 1129 (85%) children were laboratory-confirmed to have measles virus infection and resided within one of 68 townships in Lusaka. Of the confirmed cases, 514 (46%) were male and the median age was 14 months (IQR = 9, 37), with a minimum of 2 and maximum 215 months (15.5 years). Only 209 (19%) had a history of measles vaccination, with 357 (32%) of unknown vaccination status. HIV-infection was confirmed in 164 children (15%) (Table 1). Temporal variation in the number of children studied was apparent in the time-series (Figure 1), reflecting the seasonality of measles virus transmission in Zambia prior to the SIA in 2003.

**Table 1:** **Characteristics of measles cases during the endemic (1998-2002) and epidemic (2010) periods in Zambia**

**Variable**	**1998-2002**	**2010**
	**Median (25** ^**th**^ **percentile, 75** ^**th**^ **percentile)**	**Median (25** ^**th**^ **percentile, 75** ^**th**^ **percentile)**
Median age in months (IQR)	14 (9, 37)	12 (7, 29)
Male	613 (54%)	588 (52%)
Measles vaccination status		
Not Vaccinated	563 (50%)	618 (55%)
Vaccinated	209 (19%)	221 (20%)
Unknown	357 (32%)	286 (25%)
HIV infection status		
Not infected	931 (84%)	905 (80%)
Infected	164 (15%)	41 (4%)
Exposed	NA	100 (9%)
Unknown	11 (1%)	80 (7%)

**Note:** NA denotes “Not Applicable” in that during the 1998-2002 measles endemic period the HIV infection status of children admitted to University Teaching Hospital, Lusaka, Zambia was not available.

**Figure 2:**
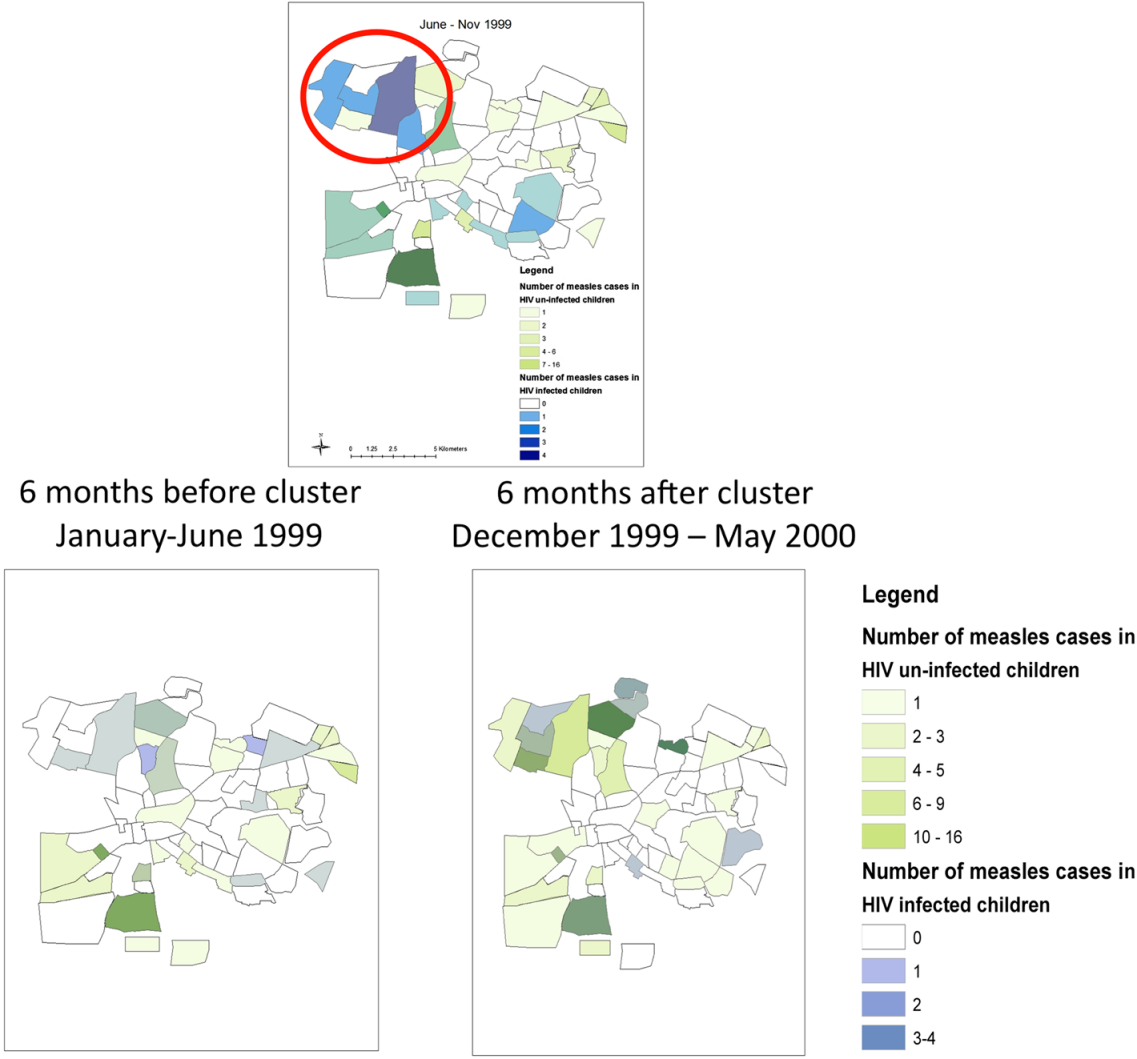
**Geographic cluster of measles cases among HIV-infected children between June and November 1999, and 6 month time periods before and after cluster.**

The space-time analysis detected a statistically significant cluster of six contiguous townships with a combined ratio of observed to expected number of measles cases in HIV-infected children of 6.4 (P = 0.03). This cluster of measles cases among HIV-infected children occurred between June 9, 1999 and November 2, 1999, a period of low transmission during which a subnational SIA was conducted in Lusaka (August 1999). During the identified cluster, 84 children with confirmed measles were hospitalized at UTH and enrolled, 17 of whom were HIV-infected (20%). Within the townships forming the spatial cluster, nine children with confirmed measles were enrolled during the interval from May to November 1999, seven of whom were HIV-infected (Figure 2). In contrast, thirteen children residing within the identified cluster were enrolled six months earlier but only two were HIV-infected, and twenty-nine children residing within the identified cluster were enrolled six months later but only three were HIV-infected (Figure 2). Thus, a higher than expected proportion of measles cases occurred among HIV-infected children in the cluster.

### 2010 measles outbreak

**Figure 3:**
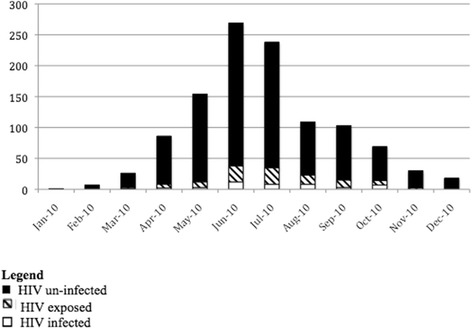
**Time-series of hospitalized children with measles, Lusaka, Zambia and included in the analysis of space-time clustering, 2010.**

Between January and December 2010, 1614 hospitalized children with clinical measles were admitted to the UTH. Medical records were available and abstracted for 1331 children (82%). Of these, 1134 (85%) were laboratory confirmed and resided within one of 77 townships in Lusaka. Of the confirmed cases, 588 (52%) were male and the median age was 23.9 months (interquartile range = (7, 29), with a minimum of 0.75 and maximum of 175 months (15 years). Only 221 (20%) had a history of measles vaccination, with 286 (25%) of unknown measles vaccination status. One hundred children (9%) were classified as HIV-exposed and 41 (4%) as HIV-infected (Table 1). The highest number of hospitalized measles cases occurred in June and July 2010 (Figure 3).

**Figure 4:**
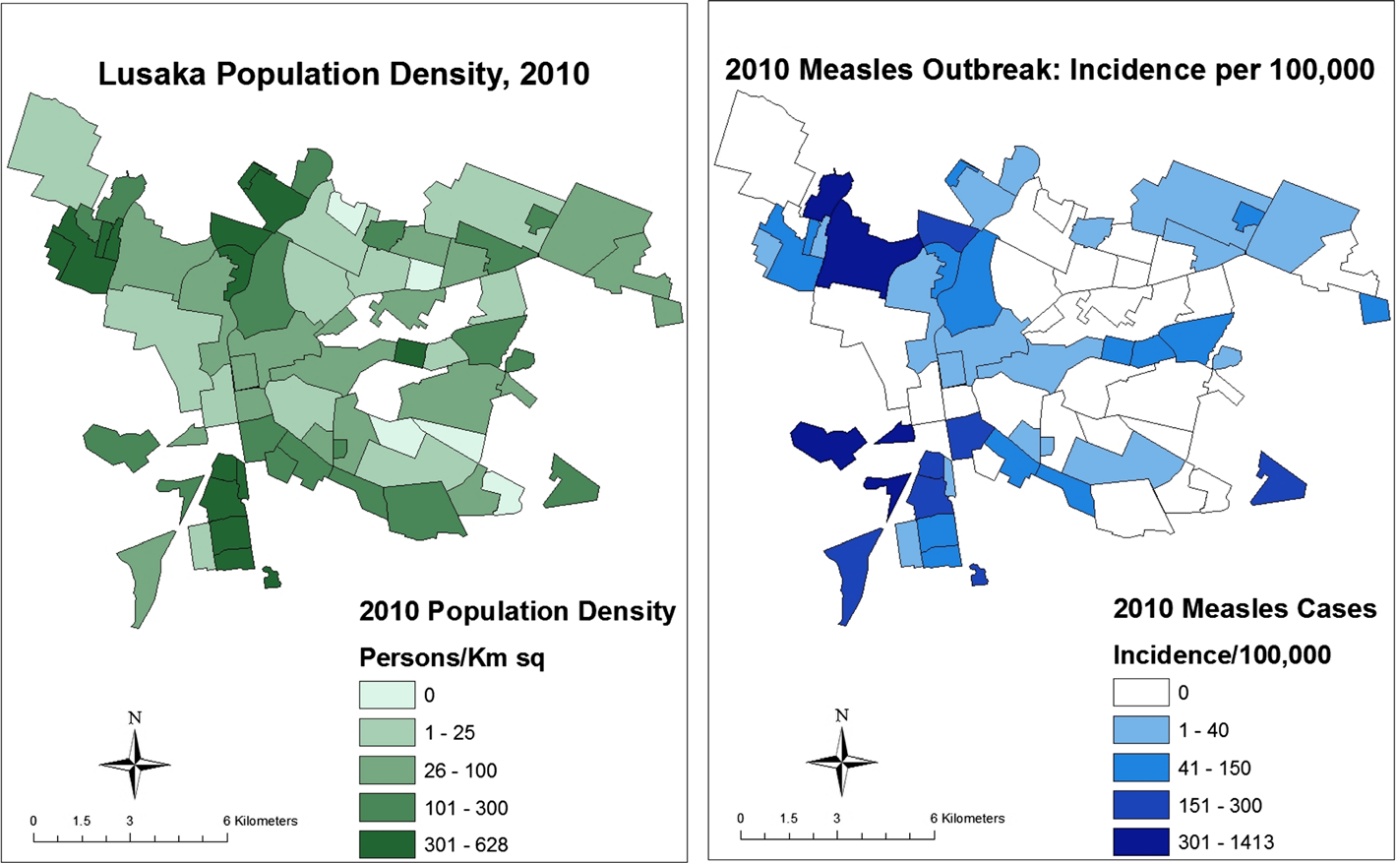
**Population density (persons per km** ^**2**^ **) and measles incidence per 100,000 persons during the 2010 measles outbreak in Lusaka, Zambia.**

**Figure 5:**
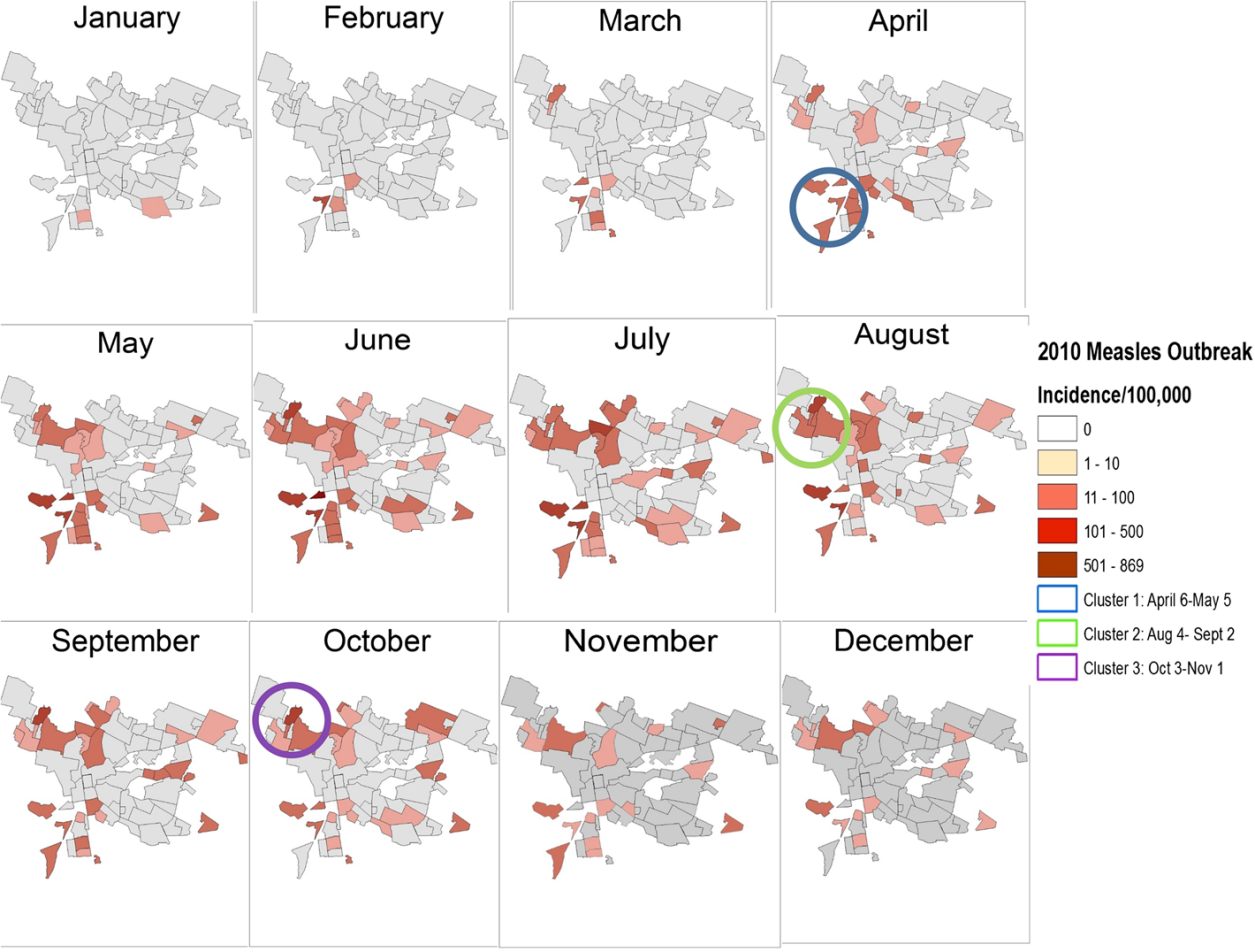
**Distribution of hospitalized children with measles in 2010 with identified clusters.**

The spatial distribution of the incidence of measles cases per 100000 persons in 2010 resembled the spatial pattern of population density: townships with higher population density had a higher overall reported incidence of measles in 2010 (Figure 4). No statistically significant clusters of measles cases were identified among HIV-infected or exposed children. However, three statistically significant and temporally distinct clusters of measles cases were identified using the space-time permutation, in April (P < 0.0001), August (P = 0.002) and October (P = 0.01) in three different geographical sections of the city (Figure 5). There were no significant differences between the three spatial-temporal clusters with regards to age, sex, HIV infection status or measles vaccination status among children with measles (Table 2).

**Table 2:** **Characteristics of measles cases within and outside each space-time cluster identified during the 2010 measles outbreak in Lusaka**

**Variable**	**Time Period 1: April 6-May 5**		**Time period 2: Aug 4- Sept 2**		**Time period 3: Oct 3-Nov 1**		**Total**
	**Cluster 1**	**Outside cluster 1**	**Cluster 2**	**Outside cluster 2**	**Cluster 3**	**Outside cluster 3**	**All cases 2010**
Number of Cases	62	36	58	54	17	49	1,134
% Male	42%	44%	40%	54%	53%	49%	52%
Age in months (median, IQR)	13 (7,32)	24 (12,55)	9 (6, 57)	9 (6, 33)	12 (7,64)	13 (7,43)	12 (7,29)
HIV infection status							
Infected	2 (3%)	0 (0%)	1 (2%)	6 (11%)	3 (18%)	3 (6%)	41 (4%)
Exposed	4 (7%)	3 (8%)	10 (18%)	6 (11%)	2 (12%)	5 (10%)	100 (9%)
Not infected	51 (84%)	33 (92%)	39 (70%)	38 (72%)	11 (65%)	33 (67%)	905 (80%)
Unknown	4 (7%)	0 (0%)	6 (11%)	3 (6%)	1 (6%)	8 (16%)	80 (7%)
Measles vaccination status							
Vaccinated	16 (26%)	8 (22%)	10 (18%)	8 (15%)	2 (12%)	8 (16%)	221 (20%)
Not Vaccinated	36 (58%)	21 (58%)	31 (54%)	31 (59%)	8 (47%)	25 (51%)	618 (55%)
Unknown	10 (16%)	7 (19%)	16 (28%)	14 (26%)	7 (41%)	16 (33%)	286 (25%)

## Discussion

Measles cases were spatially and temporally clustered during a large outbreak in Lusaka, Zambia in 2010, reflecting an evolving epidemic of local transmission foci and suggesting the potential to intervene through targeted vaccination efforts. Clustering of measles cases among HIV-infected children was identified prior to the introduction of intensive measles control efforts and during a period between seasonal peaks in measles incidence, suggesting susceptible HIV-infected children could contribute to measles virus transmission during inter-epidemic periods. Densely populated urban centers may pose an obstacle to measles control and elimination because the high levels of population immunity necessary to interrupt measles virus transmission are difficult to achieve [5]. Mathematical models of meta-populations, accounting for local communities or patches, further confirm that heterogeneities in immunity can lead to increased rates of infection among susceptible individuals [29].

HIV infection is unlikely to be randomly distributed within urban communities, but rather concentrated in high-risk populations. In these urban pockets, HIV-infected children may remain susceptible to measles despite vaccination and antiretroviral therapy, facilitating measles virus transmission [6]. HIV-infected children may contribute to measles virus transmission in urban settings with high HIV prevalence particularly during inter-epidemic periods when the number of susceptible children is depleted. No published studies have examined measles clustering among HIV-infected persons in sub-Saharan Africa.

There are several limitations to this study. The first is the bias of using hospitalized cases. The use of hospital-based data may not be representative as not all children with measles residing in Lusaka were likely hospitalized at the UTH during either study period. A mathematical model was used previously to estimate the probability of hospitalization for measles in Lusaka, Zambia, which was 6% for children younger than 1 year of age and 1.2% for persons older than 5 years of age [14]. Although some cases may have been referred to smaller health centers, UTH is the largest public hospital in Lusaka and likely received most of the cases requiring hospitalization. Nevertheless, we believe these biases in the study population were unlikely to result in the space-time clustering of measles cases among HIV-infected children. The second is the small proportion of cases with confirmed HIV infection in 2010. This may be because HIV infection in 2010 was established from medical records. However, the University Teaching Hospital Pediatrics and Child Health wing has a well-established opt-out, routine HIV testing service [30] for all hospitalized children. The observed small proportion of confirmed HIV-infected children with measles in 2010 could be a reflection of the declining incidence of HIV infection resulting from the programs to prevent mother-to-child transmission introduced over the past decade in Lusaka [31,32]. The spatial point pattern based on the geolocation of each child’s residence could not be analyzed as only the township was recorded, with loss of spatial accuracy. Lastly, to calculate incidence we used the total population per township as the denominator and not the total number of children. If the proportion of children within each township varied, this heterogeneity may bias our rates. As a consequence of the case–control study design, as well as no data on the number of HIV-infected children within each township, we were not able to measure the incidence of measles in HIV-infected and uninfected children.

Additional data from outbreak investigations would help assess the magnitude of clustering and the distribution of measles clustering in cases not identified at UTH. Mapped spatial data of HIV and measles incidence at the township level would be useful to further quantify the degree of clustering of measles.

## Conclusions

We demonstrated space-time clustering of measles cases during an outbreak in a densely populated urban center in sub-Saharan Africa and identified a cluster of HIV co-infected children during an earlier endemic period. Prediction and early identification of spatial clusters of measles outbreaks will be critical to achieving measles elimination and HIV infection may contribute to spatial clustering of measles cases in some epidemiological settings.
